# The Criminal Himself

**Published:** 1902-10

**Authors:** S. G. C. Pinckney

**Affiliations:** Atlanta, Ga.


### THE CRIMINAL HIMSELF.*

BY S. G. C. PINCKNEY, M.D.,

ATLANTA, GA.

In considering the complicated problem of crime and its perpetrator, the criminal, we must from the commencement speak with most guarded reserve. In even the high civilization of to-day the idea that the criminal is anything but an arrant and voluntary rogue is hooted at. The outlaw theory is still the dominant one. To the lay mind crime is a voluntary act without adequate cause, save a natural, restrainable and willful viciousness. The criminal is one who sets aside the laws of society through sheer perverseness, or for personal gain. In primitive society such views were perhaps justified. They were at any rate not particularly harmful. In the struggle for existence crime as crime was a very uncertain quantity. Laws were lax. Money compensation or direct revenge on the perpetrator gave complete satisfaction.

At the present time it is scarcely worth while to even consider the then existing penal code, save as a matter of historical interest. Even down to such a late time as the eighteenth century the position of the criminal had not changed much. An outcast from society, the penalty of his act had scant or no relation to the damage done.

When we look at the penalties suffered in England under the Georges, we are to-day horrified to find such acts as petty larceny

*Read before the Georgia Sociological Society, Atlanta, June, 1902.

punished by death, to read of wholesale lopping off of hands and feet, and to find even burning resorted to. The very fact of the inadequacy of the crime as related to the punishment striking us so strongly should be a proof to ourselves that we mentally balance one against the other. And yet in spite of the severities dealt out crime did not decrease. Emotional executions were attended by robberies in the very crowds gathered to see them. The confessions of condemned criminals hawked about the streets were promptly followed by fresh robberies and murders. Criminality became rampant, increasing beyond all natural ratio, increasing to such an extent that England, frankly admitting its inability to cope with such extension, found it best to place her convicts in penal colonies.

Australia, first found in 1770, was utilized eighteen years later as a convict settlement. In the following fifty years some eighty thousand malefactors were transported there, transported under the most unfavorable auspices, in filthy ships, with uneatable food, placed in a country without one trace of civilization, there to be regarded as a vicious animal that had righteously been thrust forth from the better members of society. Hard and irrational as this must seem from the humanitarian standpoint to-day, unequal as the penalty of utter isolation from civilization was to the degree of the crime, it was still the great saving act. England, unconsciously, had found a solution of her criminal problem, a solution that we cannot better to-day, a solution that at this moment has been reached through deductive reasoning by the foremost criminal anthropologists. To-day a vast area of the world's surface, considered at that time but as a haunt for the dissolute and degenerate, is a thriving and prosperous continent, having a population of the highest grade of refinement and culture, demanding the best materials produced in the manufacturing world, and paying for them with funds inherited or earned by the children most frequently of original convicts, once considered the scum and scourge of England.

In five years more a most remarkable thing had happened. Respectable settlers had immigrated, perhaps not in very large numbers, but still sufficient to show a spirit of law and order. Many of the convicts had been liberated in the surrounding country on ticket of leave. Property had been acquired, and its possession jealously guarded. In five short years the respectable element so enormously predominated, not through immigration, but rather from the awakening of moral ideas in the convicts themselves, that crime was the unusual, and they had installed a constabulary force of one thousand men to keep order. In five short years, in a semi-wilderness, peopled by the acknowledged outcasts of society, the ethical, moral, and legal conscience had risen to such a plane as to consider crime *crime*, and to demand protection of individual rights. Society to the very largest extent had rejuvenated itself, and was starting the world anew. More than that, so repellant had acts of violence to property and person become, that England was forced to discontinue her transportation of convicts, because the convicts themselves—now landed proprietors, artisans, and even financiers—entered their protest. They put in their claim that years of honesty, work, and frugality was a sufficient plea for their rehabilitation, and entitled them to the right of exemption from fresh contamination. Pondering over their own past, remembering their own temptations, can we blame them for eagerly begging protection for their children ? In these few years they had become law-abiding citizens, with the same dread and horror of the criminal that society once had of them. I have used England as an example here simply because its history could be the most readily investigated. In a century and a half we have had an opportunity to observe the results obtained in an entirely new departure in the treatment of the criminal, and the ultimate results. Let us place that criminal or his descendants on an equality with ourselves.

England and the colonists had no knowledge of sociology as an art, did not differentiate the occasional from the habitual criminal. There was neither rhyme nor reason for a colonization save the expediency of getting rid of a dangerous part of the population ; yet the results were enormous.

It would seem the fate of new countries to be settled by the vagrant classes, first by adventurers and gold-seekers, next to be the dumping-ground for the refuse of older civilization. Yet who can say that the younger countries are not the stronger, perhaps by reason of this very fact? America has passed through these regulation stages, with the saving clause that the criminals imported here were nearly always of a political type. The redemptioners appear to have been rather above the ordinary grade of intelligence, and were simply those worsted in the fight for existence. After the defeat of Monmouth, a large number of his adherents were sold to the colonies. They could not in the least be considered as criminals. Belonging to the peasant class, they readily assimilated with the population, and became the small farmers of Virginia and the Carolinas. Nothing but the moral stigma of treason could attach to them, and this, in a country three thousand miles away, with such an unpopular monarch as James II., could mean but little. The paucity of gold found, a lack of material for immediate barter, kept out the professional wealth-accumulator. None but the hardiest and most adventuresome could hold a lodgment in our continent. Colony after colony was annihilated by pestilence, famine, and the Indian. Those who survived had an inherent dominance that must have been immensely valuable to the young State. In the North, in the New England States, dogmatic Puritanism held undisputed sway. Morals were supposedly of the highest class; fines and penalties for their non-observance were very strict. Morality was at a premium, and crime almost unknown.

Our own State of Georgia was primarily settled almost as a philanthropic movement. A knowledge of the antecedents and the character of the settler was required, and the question as to whether his misfortunes were the result of circumstances or of his personal immorality was carefully weighed. So far as elimination of the undesirable could be carried out, it was.

Apparently, in the settlement of the original colonies the undesirable element was eliminated just so far as was possible, the adventurers by the Indian, the wealth-seekers by the lack of accessible plunder, the criminals by the highly religious and Puritanical public sentiment. Our start as a Nation could scarcely have been more favorable. We had little to undo and much solid material to work with. Healthy growth was implied from the beginning. During our earlier life the exigencies of existence fostered this national health. The progression of colonization being rapid, settlements were sparsely populated. The necessities of mutual protection were so great that neighbors were friends, and friends almost stood in the light of relatives. The vices of older civilization had no place in communities where the necessities of life were all that could be hoped for. To be sure, minor lapses in morals were frequent, but the greater crimes were almost absent. Simplicity was an essential, a necessity, and with the simplicity of life, of food, and of manners, we have a simplicity of mentality. It is doubtful if anywhere in the world's history there can be found a more honest and less inherently criminal population than the colonists of America during the earlier part of the eighteenth century. It is to be remembered that at this time the life of the colonist was an active outdoor one. He was little given to the introspective, had small reason for personal display, and his claims of superiority were based more on personal prowess than the possession of worldly goods. Therefore the ordinary incentives to crime were lacking. He was scarcely liable to kill his neighbor when he might need that neighbor to assist in the protection of his own family from the savages. He was not liable to steal, when he had no occasion for adornment, and his frugal habits, coupled with a natural egotism as regards his personal powers, made the procuring of a livelihood comparatively easy.

On these contrasting continents it must be admitted that the odds were all in favor of America. Settled as it was by a naturally hardy part of the population, most generally by the county element, by those who accepted danger and toil by preference, those of strong religious convictions settling primarily in small colonies, in which religion and its ministers were accepted as authorities even higher than the civil laws, it would seem that the progressive degenerative criminality, if not already eliminated, would in a few years be obliterated. Australia with its convict population, drawn principally from cities, the slums of England, was identically of the same stock, which seems to have poor chances, and yet to-day the criminal ratio is about the same. At no time can we obtain from the records that there was very much difference. What evening process had nature devised or mankind worked out to account for this? Like other neurologists and alienists, I have been unable to accept any but the modern views of criminology. The elaborate investigations, the convincing proofs, the mental honesty and disinterestedness of many men, who engage in the consideration of the prevention of crime, must attract the attention of the most thoughtless.

During my work in medicine it has been my fate to be personally called upon to pass judgment on the mental caliber of many criminals, more especially those accused of the gravest crimes. This has of necessity compelled a serious consideration of the subject, and the greater the consideration the more conservative I have found myself growing. Let us once and for all dismiss the occasional criminal from our consideration here. He is not pathological. He is simply a creature of circumstances. So long as the necessity for food and protection exists, he will exist. Improved conditions of living, better co-operative measures between labor and capital in all our industries, will gradually solve his problem to a great extent. The subject is so vast that I will not even attempt to touch it at present.

But the habitual, the degenerate criminal is a subject for the deepest consideration. It is to this type that the investigator has given his time. It was only to be supposed that the ratio of degeneracy would increase normally with the population; that individuals coming from unsound stock, born in families of poor physical or mental make-up, inheriting such degenerative diseases as hereditary tuberculosis, syphilis, chronic insanity, and the like, would from the very lack of their inhibitory powers procreate at least as rapidly as the healthy stock. Even granting a high infant mortality, statistics show that their birth-rate is even more in proportion from this very lack of self-control than in healthy individuals. Emotional, thinking little of the future, without the ordinary restraints of prudence that govern mankind, they marry early, and, if not married, supply by far the largest percentage of illegitimate children. Even allowing for this natural increase, degeneracy, as we can trace it in medicine, has gone beyond all bounds. No principle of natural progression will explain the doubling of the percentage of insane to sane every ten years as has occurred in some of our States.

That new elements in the strain of life are appearing with each generation is undoubted. This is one of the many points we are apt to forget, that out of to-day's healthy stock in many ways we are producing the degenerate of the future. In the elaborate investigations, which it is now the fashion to carry on, but comparatively few points are brought out in regard to this prevention of degeneracy. Alcohol, as a causative factor, offers such vast possibilities that it seems to absorb the attention of the investigators. The stress of life, the difficulty of procuring a livelihood, are brushed aside as necessities of civilization that we are unable to combat. The prevention of marriage of degenerates has been taken up in a sporadic, half-hearted way. Bills have even been introduced in the Legislatures of several States looking to this end, only to meet with derision. From the practical standpoint, the view at present is to wait till man becomes a degenerate, then deal with him. In fact, until the last few years all efforts to check crime were in the nature of retaliation or physical restraint. Since the investigations, more especially of the Italian schools, have been carried on the subject of degeneracy has received its due attention. The habitual criminal has been posed in the same group with the insane and the weak-minded. More and more has he been relegated to the ranks of the diseased. His incurability has been insisted upon; he was predestined, doomed even before birth to the life he should lead. It is easy in many ways to prove this. Even physical signs are so apparent. The stigmata are held up as evidence. When you can show that only 4 per cent. of normal individuals have five or more peculiar physical blemishes, while 27 per cent. of criminals have the same number, it would almost seem as though nature had allowed us to pick out the dangerous element in society. The alliance between crime and other types of degeneracy is again reinforced by the presence of a large proportion of the epileptic and the insane, who also show these stigmata, all above 25 per cent.

The criminal himself is therefore, in our modern view, a hopeless proposition, so far as he or his immediate descendants are concerned. Yet one point has not had its due prominence, and that is that the increase of crime is far below that of degeneracy, as shown in other types. If the degenerate may, as chance wills it or circumstances demand, be either a criminal, epileptic or an insane, how does it happen that the increase in the latter types is so infinitely greater than in the former? Can any State show an increase of 100 per cent. in proportion to the ratio of population of its criminals in ten years? Are the immediate determining factors, such as stress of life, alcohol, insufficient food, and the like, more prone to produce the insane than the criminal? We should think not. In degenerates assuredly the most typical of all characteristics is the lack of self-control. We can hardly credit them with starving themselves into insanity rather than stealing food; of denying themselves the necessities or luxuries of life rather than acquiring them by unfair means. Such determining factors as would direct the degenerate into criminality are as much present as they ever were; and yet, strange to say, the degenerate is yearly becoming more respectable.

Has our more efficient protective system anything to do with holding down criminal propensities? Certainly not enough to change statistics to this extent. The bulk of crime is to-day committed in large cities, where protection is greatest. The subject is a very curious one. The very class, who, it is insisted, are hopeless of redemption, are showing us themselves the fallacy of these views. Is any higher ethical element entering into the ranks? I think none of us would give the habitual criminal credit for much moral reasoning. It is very much of a puzzle, the same puzzle we have to solve when we look back at Australia's regeneration.

Seemingly, degeneracy, as shown in criminality, advances so far, then ceases to progress. As shown in all other types, its increase is out of all proportion. Is there any species of natural selection in degenerates that would determine the type it should take? Apparently not. It is customary to look upon the insane as individuals often one step removed from mental brilliancy, upon the criminal as of a low mental organization. I have been unable to find this difference. Their past histories generally give about an equal mental equation. Some process is going on, either in society or in the degenerate, that is altering things remarkably for the better. Not one, but undoubtedly many agencies, are carrying on this evening process that changed the vast convict settlement in Australia into law-abiding citizens, with no higher percentage of criminals than resulted from the small leaven that produced our present crime in America.

I have no explanation to offer of this anomalous condition. All rational reasoning, if we start with the hypothesis that the criminal is born what he is, must fail us. It would seem that we can only believe that either degeneracy is being gradually wiped out; that ethical, religious and physical powers are restraining the spread of crime, or that the criminal is regenerating himself. That degeneracy is increasing is shown by its spread in other directions; that ethical ideas, though restraining certain border-line cases, could not have much effect upon a real physical defect, is also apparent. This brings us back to the criminal as the prime factor in the change. There is no doubt that there is a gradual regeneration going on among the greater number of degenerates, just as tubercular families of a few generation ago now show healthy descendants. We will find that the degenerates of the same time are now giving us many brilliant members of society. The lopping off of the weaker members, intermarriage with healthy stock, and more temperate modes of life have done much to strengthen the nervous system.

This very fact of healthy intermarriage, with perhaps the accumulation of a small amount of the world's goods, has undoubtedly had a more restraining influence on criminality than on any other type of degeneracy. The possession of personal effects places the criminal to a certain extent among the law-abiding class. For the first time he can see the advantages of the law. His point of view is quite changed. Such latent possibilities of regeneration as exist in him are brought out. Undoubtedly this was one of the chief influences in Australia. The hopelessness of the future, dependent upon preying upon others, becomes less pronounced. The possibilities of more accumulation become greater to him. Certainly but a moderate proportion of degenerates show active symptoms. A small weight in the balance on either side may determine the entire future. He instinctively shuns past associates, whom he now considers of an inferior grade to himself, and, as in Australia, holds as much beneath him the hopelessly incorrigible. In addition to this we had in England's penal settlement the immense advantage of an out-of-door life with its attendant increase in nutrition and lack of emotional excitement. Such conditions would have less effect in restraining the other types of degeneracy, they being unfitted to obtain these good results from either physical or mental weakness.

In America I have no doubt that our present percentage of crime has been much due to the enormous absorption required of us of low-grade foreign population. None but a virgin country of strong moral backbone could have withstood this incubus. We could not, as England did, turn them loose with such immediate prospects of building up a livelihood. We had to reconcile opposing races and religions, always a most difficult feat. When the present immigration set in, we were already well settled and prosperous. The same temptations existed here that existed in their mother countries. Large cities absorbed the greater part of the lower classes. The degenerate criminal instinctively remained there. He had but changed his locality, not his environment. Such emigrants as penetrated into the country and settled up our Western lands formed just such law-abiding communities as are to be found in Australia. Their pauperism was soon relieved by work and effort.

In this outlined sketch the only deductions to be made are that, so far as we can see, two continents starting under very dissimilar circumstances have arrived at about the same results in their criminal records. One started under favorable auspices with 300 years of civilization, and the other with unfavorable auspices with 125 years of civilization.

Surely, if criminality is but a type of degeneracy, unamenable to treatment, except as the cases die off or are restrained from propagating, some greater discrepancy would occur, some such discrepancy as appears in the other equivalents of degeneracy. Both States have been progressing in the material and intellectual advancements of the age. Australia is undoubtedly the leading dependency for the intellectual, financial, and moral worth of England. America is fast becoming the leading country of the world.

It is evident that innate restraining forces in the criminal himself are coming to the top. I take a hopeful view of the situation. Granting his degeneracy, granting his inherently bad heredity, public opinion, moral and ethical ideas, the possibilities of social regeneration, and the acquirement and possession of effects, seem slowly but surely to be acting as a stimulus towards a better mode of living. The absence of progression in percentage of crime in comparison to other types of degeneracy can only imply a rehabilitation of a vast number of what we have seen fit to call the habitual criminal. And to bear this out we find that the decrease of crime is in direct proportion as we allow the criminal a chance to better himself, as we foster his self-respect, and place him in such a situation as that he will approach the normal man. It would seem that our so-called degenerate criminality was in a great measure as competent of reformation as other curable diseases; that the healthy physical and mental life would have just as retarding an influence upon it as upon the spread of hereditary tuberculosis.

In this paper I have only endeavored to suggest some lines of thought. Conclusions and remedies for the distemper will take many years to evolve. Yet surely it will be through such mental stimulation and stirring up as occur from meetings of societies like this we are attending, that a due interest in the working-out of the problem will ensue. I am sure we, none of us, claim an ultimate knowledge of any point that we bring up here. We are simply asking ourselves certain questions, propounding certain problems, and begging the world for its own good that they help us solve them. Granting enormous resources for study, granting the clear mental insight of our great investigators, we are yet not called upon to blindly agree to a certain dictum when so much can be said on the opposite side. That the hereditary degenerate may be, and often is, a criminal, and morally irresponsible, I admit with all my heart; that he is in all cases hopeless seems to me far from true.

